# The association between parent–child and peer relationship quality in adolescence and intimate partner relationship quality in young adulthood: A two‐cohort longitudinal investigation

**DOI:** 10.1111/jora.70184

**Published:** 2026-05-08

**Authors:** Khya Marabel‐Whitburn, Emma M. Marshall, Craig A. Olsson, Jacqui A. Macdonald, Elizabeth A. Spry, Stephanie R. Aarsman, Primrose Letcher, Tina Kretschmer, Christopher J. Greenwood

**Affiliations:** ^1^ Deakin Lifespan Institute, School of Psychology, Faculty of Health, Deakin University Burwood Victoria Australia; ^2^ Murdoch Children's Research Institute, Royal Children's Hospital Melbourne Victoria Australia; ^3^ Department of Paediatrics Royal Children's Hospital, University of Melbourne Melbourne Victoria Australia; ^4^ Institute of Psychology, Friedrich‐Alexander University Erlangen‐Nuremberg Erlangen Germany

**Keywords:** adolescence, cohort study, intimate partner relationship, parent, peer, target trial emulation

## Abstract

Adolescent experiences in close relationships play a seminal role in social–emotional development across the lifespan. Here, we examine the unique and combined effects of parent–child and peer relationship quality in adolescence (age 11–13 years) in shaping intimate partner relationship quality in young adulthood (age 19–28 years). Prospective data were from the Australian Temperament Project (*n* = 1117) and the TRacking Adolescents' Individual Lives Survey (*n* = 1512). Higher parent–child and peer relationship quality were associated with higher intimate partner relationship quality (*β*_range_ = 0.07–0.12), an effect that was strongest when both parent–child and peer relationships were of high quality (*β*_range_ = 0.13–0.19). No sex differences emerged. Results were consistent across both the ATP and TRAILS cohorts. Promoting healthy parent–child and peer relationships in early adolescence may help strengthen intimate partner relationships in young adulthood.

## INTRODUCTION

A person's subjective perception that their intimate partner relationship is good (vs. bad), referred to here as high relationship quality (Finkel et al., 2017; Joel et al., 2020), has a range of physical and mental health benefits including better well‐being, cardiovascular health, sleep, physical activity, and longevity (Holt‐Lunstad et al., 2010; Kiecolt‐Glaser & Wilson, 2017; Proulx et al., 2007; Robles et al., 2014; Roth et al., 2025). Moreover, these benefits are comparable to those associated with other well‐known and modifiable health risk factors, such as diet and physical activity (Robles et al., 2014). Improving the quality of intimate partner relationships is therefore, an important public health priority in many countries around the world (Holt‐Lunstad et al., 2017; World Health Organisation, 2025). To do this effectively, however, one must first consider the developmental origins of intimate partner relationship quality. Young adulthood often marks the initiation and/or consolidation of long‐lasting adult intimate partner relationships (i.e., de facto and/or marital relationships; Arnett, 2023; Shulman & Connolly, 2013), yet, the foundations for these relationships are established even earlier (i.e., in adolescence; Collins et al., 2009).

The developmental cascades model offers a useful framework for understanding how strengths and vulnerabilities in earlier life domains can scaffold and accumulate over time, and set in motion pathways that shape future development (Masten & Cicchetti, 2010; Van de Bongardt et al., 2015). When applied to the context of social relationships, we would expect that foundational relational experiences with parents and peers cascade forward, predicting the quality of future intimate partner relationships that occur later in life. This perspective aligns with life course perspectives, which emphasize that adult relationship quality reflects the cumulative influence of earlier social experiences and developmental processes (Elder, 1998; Elder et al., 2015). Despite this, few studies are able to prospectively map relational pathways across the life course, limiting our understanding of how these early close relationships cascade over distinct developmental periods.

### Close relationships in adolescence

Early adolescence, in particular, is a watershed period of social–emotional development characterized by greater independence and autonomy that bridges childhood and adulthood (Collins & Madsen, 2006). Hallmarked by a drive for greater independence, adolescence brings both psychological *and* social change, where relationships and social interactions become more diffuse, salient, and meaningful (Collins & Laursen, 2004; Main et al., 2025). This period is also particularly critical for relational development, with shifts in social and emotional functioning laying the foundation for trajectories that shape later relational outcomes (Dahl et al., 2018). This occurs as adolescents begin to explore relationships outside the family home—most notably peer relationships (Brown & Larson, 2009; Collins & Steinberg, 2006).

Peer relationships play a crucial role in adolescence fostering independence and the development of new identities beyond the family environment (Branje, 2022; Collins & Laursen, 2004; Sebastian et al., 2008). Critically, these relationships also serve as a training ground for exploring and cultivating romantic relationships, often providing the foundation for early romantic experiences (Furman & Rose, 2015; Van de Bongardt et al., 2015). These relationships also help develop the relational skills needed for high‐quality intimate partner relationships, including capacities to communicate effectively, build intimacy, navigate disagreements and conflict, and engage in responsive support exchanges (Allen et al., 2020; Collins et al., 2009; Furman & Rose, 2015).

As this shift occurs, relationships with parents undergo significant changes, often becoming marked with an increase in conflict and decreased feelings of closeness (Laursen & Collins, 2009). Despite these shifts, the parent–child relationship continues to play an important role in social–emotional development during adolescence in two key ways. First, parent–child attachment processes shape internal working models (mental representations of the self and others) that guide expectations and behaviors in close relationships (Bowlby, 1982; Dykas & Cassidy, 2011; Laursen & Collins, 2009). Second, and akin to peer relationships, adolescent–parent relationships provide a crucial context for relational skill development. Indeed, they allow the adolescent to develop effective communication, emotional regulation, and conflict resolution skills, which are critical for forming and sustaining high‐quality intimate partner relationships in young adulthood (Ford et al., 2023; Kretschmer et al., 2017; Ratliff et al., 2023; Steinberg, 2001; Tan et al., 2016).

Empirical evidence has revealed a consistent, positive, prospective association between adolescent parent–child relationship quality (e.g., secure attachment and quality) and intimate partner relationship quality (Ford et al., 2023; Rauer et al., 2013; River et al., 2022; Roisman et al., 2009; Schulz et al., 2023). Of particular relevance, Kretschmer et al. (2017) found a positive association between adolescents' reported positivity in parent–child relationships (age 11 years) and intimate partner commitment and satisfaction in young adulthood (age 22 years). Extending this evidence, Ford et al. (2023) found that adolescents who reported more warm, supportive, and communicative relationships with both mothers and fathers (ages 12–17 years) subsequently reported higher quality romantic relationships in young adulthood (ages 24–32 years). Less prospective research, however, has examined the association between adolescent peer relationships and intimate partner relationships in young adulthood. The findings are also more inconsistent. To illustrate, Allen et al. (2020) found a positive association between several peer relational factors (i.e., assertiveness, social competence, formation, and maintenance of close peer relationships) during adolescence (age 13–18 years) and intimate partner relationship satisfaction during the later periods of young adulthood (age 27–30 years), when adjusting for demographic characteristics, adolescent depressive symptoms, and baseline romantic satisfaction. However, when accounting for family dynamics, personality traits, and emotion regulation, Seiffge‐Krenke et al. (2001) found a negligible association between adolescents' reported intimacy and reliance in close peer relationships (age 17) and the connectedness and attraction in intimate partner relationships in the early stages of young adulthood (age 20).

While there is some empirical evidence that adolescent parent–child and peer relationship quality forecasts intimate partner relationship quality in young adulthood, limitations remain in the literature. First, the majority of current studies focus on a single follow‐up period and capturing outcomes at one discrete time point (i.e., early or late young adulthood), limiting understanding of the developmental trajectory of intimate partner relationships and how relationship patterns evolve or persist over time. Second, most of the studies fail to use methods that minimize bias within observational data to allow for stronger causal inferences. Third, very few rigorous prospective studies examining both adolescent parent–child *and* peer relationships have been conducted (see Crockett & Randall, 2006 for an exception). Thus, research is yet to systematically explore how adolescent parent–child and peer relationships *interact* to affect future intimate partner relationship quality.

### The interplay between parent–child and peer relationships

Relationships in different settings of an adolescent's life rarely operate independently of one another. Healthy relational development requires an individual to integrate the characteristics from diverse relationships into a cohesive relational ecosystem (Bronfenbrenner, 1979; Reis et al., 2000). Optimal relational health across the lifespan should, therefore, occur when an adolescent has been able to cohesively integrate their close relationship experiences and foster the distinct yet complementary skills that were obtained from the high‐quality close relationships that exist within their social network (Collins & Sroufe, 1999; Furman et al., 2002; Van de Bongardt et al., 2015). At the most polarized level, developmental outcomes are likely to differ markedly among young people who report high‐quality relationships with parents and peers compared to those who experience poor quality relationships with parents and peers. However, situations in which an adolescent has been able to experience and foster healthy relationships in one context but not the other may also plausibly leave some relational skills underdeveloped and lead to a less cohesive ecosystem.

Empirical research, however, has not been able to identify an association between adolescent parent–child and peer relationships in combination and intimate partner relationship quality in young adulthood. This is largely due to the lack of prospective studies examining both parent–child and peer relationship quality. One prospective study, however, found no evidence for an interaction between parent–child and peer relationship quality at age 16 and the association with intimate partner relationship quality at age 23 (Crockett & Randall, 2006). Nevertheless, replication is necessary in light of more recent research, which has found that adolescent parent–child and peer relationships interact to predict other important and associated developmental outcomes in adulthood, such as mental health and the likelihood of having children (Macdonald et al., 2021).

### Current study

The current study utilizes rare prospective data from two long‐running studies of social and emotional development in Australia and the Netherlands. Both countries have well‐established aligning longitudinal studies of social development and intimate relationships, enabling us to replicate the findings within two samples, check the robustness of the results across two countries, and enhance the generalizability of our findings. The aims are threefold: (1) to estimate the causal effect of parent–child and peer relationship quality in adolescence (ages 11–14) on young adult intimate partner relationships (ages 19–28 years), (2) to examine whether the effects of adolescent parent–child and peer relationships are interdependent in shaping intimate relationship quality in young adulthood, and (3) in line with current recommendations to routinely examine gender differences in developmental and health research (Heidari et al., 2016), to examine whether effects differ between male and female participants. In detail, gender socialization processes are thought to shape relationship development in distinct ways, with evidence suggesting that boys and girls are exposed to different expectations and norms regarding intimacy, autonomy, and emotional expression from early adolescence (Collins & Laursen, 2004; Feraco & Meneghetti, 2023; Moreau et al., 2019). Given these evidenced gender socialization processes, it is important to consider whether the cascading effect of close relationship quality from adolescence to young adulthood differs in strength for males and females. While this study is not preregistered, we hypothesized that high‐quality parent–child and peer relationships in adolescence would both be associated with higher intimate partner quality in young adulthood. However, we expected this association to be the strongest when high‐quality relationships were experienced with both parent *and* peers simultaneously. To minimize bias in our methodology, and to strengthen causal inferences, we used contemporary approaches: Target Trial Emulation (TTE) framework and Directed Acyclic Graphs (Hernán et al., 2022; Hernán & Robins, 2016).

The TTE framework guides the design and analysis of observational studies to mimic the structure of randomized controlled trials, thereby improving causal inference (Hernán et al., 2022; Hernán & Robins, 2016). By specifying the components of the hypothetical trial, the framework helps to identify potential sources of bias, such as confounding and selection, and clarify the assumptions required for causal interpretation. Directed Acyclic Graphs (DAGs) were used to visually represent causal pathways between adolescent relationship quality and intimate partner relationship quality in young adulthood and identify potential confounders to control for. These approaches are particularly valuable in longitudinal studies where randomization is not feasible, and confounding is a concern.

## METHODS

### Participants and procedures

Data were sourced from two mature cohort studies: the Australian Temperament Project (ATP) from Australia and the Tracking Adolescents' Individual Lives Survey (TRAILS) from the Netherlands. The ATP has tracked the socioemotional development of a birth cohort (Generation 2, G2, *n* = 2443) and their parents (Generation 1; G1) since 1983. This has amounted to a total of 16 current waves of data collection. Participants were recruited through maternal and child health centers across Victoria, with the sample being broadly representative of the Victorian population at the time. Data were collected at regular intervals through parent and teacher reports in early waves, and later through participant self‐reports and follow‐up interviews. For more information on the ATP cohort, see Murdoch Children's Research Institute (n.d.), Olsson et al. (2022), and Prior et al. (1989). Data collection waves were variously approved by Human Research Ethics Committees at the University of Melbourne, the Australian Institute of Family Studies, and/or the Royal Children's Hospital, Melbourne.

TRAILS, one of the longest running studies in the Netherlands, has tracked social and emotional development of a cohort (Generation 2, G2, *n* = 2229) and their parents (Generation 1; G1) since 2001. TRAILS has been ongoing for nearly 25 years, totaling eight waves of data collection to date. Participants were recruited from school registers in five municipalities in the north of the Netherlands, including urban and rural areas. Parents of children provided informed consent prior to data collection. As in the ATP cohort, data were collected through a mix of parent, teacher, and self‐reports, conducted at regular follow‐ups. For more information on the TRAILS cohort, see Oldehinkel et al., 2015. Data collection was approved by the Dutch Central Committee on Research Involving Human Subjects (NL47782.042.14). Questionnaires were translated from Dutch to English for the current study.

The current study used a sample of 1117 G2 participants from the ATP and 1512 G2 participants from TRAILS who reported that they were in an intimate partner relationship in one or more of the young adult surveys (ATP: age 19–28 years; wave 13–15; TRAILS: age 19–26; waves 4–6). Both cohorts were broadly representative of their national populations in terms of socioeconomic distribution and reported strong retention across waves, enhancing confidence in the representativeness of their samples (see Table 1 for descriptives; Oldehinkel et al., 2015; Olsson et al., 2022; Prior et al., 1989). On average, just over half of the sample were female in both the ATP (*n* = 652, 58%) and TRAILS (*n* = 868, 57%). In the ATP, 66%, 81%, and 91% of participants reported being in a relationship at wave 13, 14, and 15, respectively. In TRAILS, 67%, 76%, and 83% of participants reported being in a relationship at wave 4, 5, and 6, respectively.

**TABLE 1:** Descriptive statistics of the analytic sample.

	ATP			TRAILS		
	*M* (SD)	95% CI	Missing	*M* (SD)	95% CI	Missing
Intimate partner relationship quality^a^						
19–20 years	3.54 (0.63)	(3.49, 3.59)	45%	5.78 (1.12)	(5.71, 5.86)	46%
22–24 years	3.60 (0.53)	(3.56, 3.64)	42%	6.07 (0.96)	(6.01, 6.14)	37%
25–28 years	3.72 (0.44)	(3.68, 3.75)	35%	6.21 (0.82)	(6.16, 6.26)	34%

	*n* (%)	95% CI	Missing	*n* (%)	95% CI	Missing
Adolescent relationship quality^b^						
Parent–child	533 (57%)	(54, 60%)	17%	1062 (72%)	(69, 74%)	2%
Peer	433 (47%)	(43, 50%)	17%	607 (41%)	(38, 43%)	2%
Parent–child and peer relationship quality^c^						
Low parent, low peer	247 (27%)	(24, 29%)	17%	368 (25%)	(23, 27%)	2%
High parent, low peer	250 (27%)	(24, 30%)	17%	506 (34%)	(32, 37%)	2%
Low parent, high peer	152 (16%)	(14, 19%)	17%	53 (4%)	(3, 5%)	2%
High parent, high peer	281 (30%)	(27, 33%)	17%	554 (37%)	(35, 40%)	2%
Potential confounding factors						
Parental separation	177 (16%)	(14, 19%)	3%	312 (21%)	(19, 23%)	0%
Gender (Male)	652 (58%)	(55, 61%)	0%	868 (57%)	(55, 60%)	0%
Mental health	192 (21%)	(18, 23%)	16%	119 (10%)	(8, 11%)	17%
Delinquency	216 (23%)	(21, 26%)	17%	66 (4%)	(3, 6%)	1%
Substance use	267 (29%)	(26, 32%)	17%	591 (40%)	(38, 43%)	3%
Temperament	252 (23%)	(20, 25%)	0%	28 (2%)	(2, 3%)	22%
Birth Country^d^	297 (28%)	(25, 30%)	4%	322 (22%)	(20, 24%)	1%

	*M* (SD)	95% CI	Missing	*M* (SD)	95% CI	Missing
Mother's age	28.2 (4.26)	(27.94, 28.45)	3%	29.43 (4.46)	(29.17, 29.68)	20%
Father's age	30.56 (4.86)	(30.27, 30.85)	4%	31.94 (4.55)	(31.66, 32.22)	35%
SES	0.07 (0.92)	(0.02, 0.13)	3%	0.03 (0.75)	(−0.01, 0.07)	1%

*Note*: Frequency estimates were based on nonimputed data. ATP = Australia; TRAILS = The Netherlands.

^a^ Intimate partner relationship quality was coded as: ATP (1 = “rarely/never/very untrue,” 2 = “sometimes/somewhat untrue,” 3 = “often/sometimes true,” 4 = “always/very true”) and TRAILS (1: “strongly disagree,” 2: “disagree,” 3: “somewhat disagree,” 4: “neutral,” 5: “somewhat agree,” 6: “agree,” 7: “strongly agree”).

^b^ High parent–child and peer relationship quality was derived from mean scores greater than or equal to 4 in the ATP (“seldom true”) and TRAILS (“almost always”).

^c^ Parent–child and peer relationship quality measure was coded as: 0 = Low peer and parent relationship; 1 = high parent relationship only; 3 = high peer relationship only; 4 = high peer and parent relationship.

^d^ Birth country: ATP = Not Australia; TRAILS = Not The Netherlands.

### Measures

While the ATP and TRAILS cohorts used different instruments and were administered in different languages (English in ATP, Dutch in TRAILS), we reviewed each item to ensure they captured a comparable overall assessment of relationship quality and potential confounding factors. We evaluated the face validity of the items and confirmed that they were conceptually similar across cohorts.

#### Intimate partner relationship quality in young adulthood

Intimate partner relationship quality was assessed at three young adult waves in the ATP (waves 13–15; ages 19–21, 23–24, and 27–28 years) and TRAILS (waves 4–6; ages 19–20, 22–23, and 25–26 years). In the ATP, young adults completed six items adapted from the Braiker and Kelley's Marital Conflict Scale (e.g., “My partner is loving and affectionate towards me”; Braiker & Kelley, 1979), on a 4‐point Likert scale: *Rarely/never/very untrue, sometimes/somewhat untrue, often/sometimes true, always/very true*. In TRAILS, young adults completed six items adapted from the Investment Model Scale (e.g., My relationship does a good job of fulfilling my needs for intimacy, companionship, etc.; Rusbult et al., 1998), on a 7‐point Likert scale: *strongly disagree, disagree, somewhat disagree, neutral, somewhat agree, agree, strongly agree*. For both cohorts, mean scores were derived for which higher scores indicate higher intimate partner relationship quality. Internal consistency was good to excellent in the ATP (*α* = .88–.92) and TRAILS (*α* = .73–.91).

#### Parent–child and peer relationship quality in adolescence

Parent–child and peer relationship quality was assessed at one adolescent wave in the ATP (wave 10; age 13–14 years) and TRAILS (wave 1; age 10–12 years). In the ATP, adolescents completed seven items pertaining to parent–child (e.g., “my parents respect my feelings”) and peer (e.g., “my friends respect my feelings”) relationship quality adapted from the Inventory of Peer and parent Attachment (IPPA; Armsden & Greenberg, 1987) on a 5‐point scale: *never/almost never true, seldom true, sometimes true, often true, always/almost always true*. In TRAILS, adolescents completed 17 items pertaining to parent–child (e.g., “my father/mother takes my feelings into account”) and peer (e.g., “my friends take my feelings into account”) relationship quality adapted from the Social Productions Functions Questionnaire (Ormel et al., 1997) on a 5‐point scale: *never, almost never, sometimes, almost always, always*. For both cohorts, mean scores were derived whereby higher scores indicate higher relationship quality. Internal consistency was good to excellent in the ATP (parent–child *α* = .86; peer *α* = .72) and TRAILS (parent–child *α* = .88–.92; peer *α* = .93).

### Potential confounding factors

Potential confounders were identified in line with the disjunctive cause criterion, considering variables with a plausible causal association to either the exposure, outcome, or both (VanderWeele, 2019). Potential confounders are presented in the Supporting Information (Table S1). To ensure temporal precedence and avoid inadvertently controlling for mediators within the causal pathway, confounders were primarily selected from baseline or pre‐exposure time points. These selected confounders span a range of domains and included characteristics and behaviors across the parent (i.e., birth country, separation, and age at birth of first child) and participant level (i.e., sex, mental health symptoms, delinquency, substance use, and temperament). Selection was guided by established theoretical and empirical evidence linking contextual factors (e.g., parent characteristics, socioeconomic, cultural background) and individual factors (e.g., personality, mental health, behavioral adjustment) to the development and maintenance of close relationship quality (e.g., Conger et al., 2010; Fergusson & Woodward, 1999; Joel et al., 2020; Whitton et al., 2008).

### Target trial emulation

All analyses were conducted in Stata 17 (StataCorp, 2021). Prior to our primary analyses, we conceptualized the data using a target trial emulation (TTE) framework to strengthen causal inference when analyzing observational cohort data (Hernán et al., 2022). Detailed information on this procedure is provided in the Supporting Information (Table S2) where we present the alignments between our analyses and an idealized randomized control trial, for ATP and TRAILS separately. In practice, this involved a series of structured design decisions that mirror the core elements of a randomized trial: (a) determining the eligibility at baseline (adolescents aged 10–18 from Australia for the ATP and The Netherlands for TRAILS); (b) defining the intervention strategies under comparison (adolescents assigned to high parent–child or peer relationship quality), including what constitutes the control group (adolescents assigned to low parent–child or peer relationship quality); (c) identifying baseline confounders and determining how to adjust for them to approximate exchangeability; (d) establishing the point of treatment initiation (age 13–14) and the follow‐up period (age 19–28); (e) selecting the outcomes of interest (self‐report intimate parent relationship quality); and (f) clarifying the causal contrast to be estimated. In our study, the TTE framework was implemented in conjunction with DAGs, which we used to formalize our causal assumptions and identify a minimal confounder adjustment set. Together, these steps provide the foundation for appropriate statistical analyses, help reduce common sources of bias in observational designs, particularly confounding and selection bias, and allow for stronger causal inference.

For all emulations, the exposure variables (parent–child and peer relationships) were dichotomized. Within the Target Trial Emulation framework, dichotomizing the exposure variable allows us to align the exposure contrasts with plausible intervention strategies that could be implemented in a hypothetical target trial (Table S2; Hernán et al., 2022). Cut‐off points were determined by examining the range of scores reported in the sample. High relationship quality was indicated by scores greater than or equal to 4 on a 1–5 Likert scale, in both the ATP (i.e., “often true”; parent–child: 57% [*n* = 533], peer: 47% [*n* = 433]) and TRAILS (i.e., “almost always”; parent–child: 72% [*n* = 1062], peer: 41% [*n* = 607]). This was done to ensure that both the high and low groups retained sufficient sample sizes for meaningful comparison, while classifying “high quality” as only those participants who reported experiencing mostly positive relationships (i.e., often). The remaining participants, who scored between 1 and 3, were combined to represent (comparably) low relationship quality. To verify the robustness of our findings, we conducted sensitivity analyses using the parent–child and peer relationship quality measures as continuous variables as well. A 4‐level variable was then created to represent the combination of parent–child and peer relationship quality (0 = low parent–child and peer relationship quality; 1 = high parent–child and low peer relationship quality only; 2 = low parent‐child and high peer relationship quality only; 3 = high parent–child and peer relationship quality). This variable was treated as categorical in all analyses, with the low parent–child and peer relationship quality group serving as the reference group in all models. A supplementary analysis was also conducted using the continuous parent–child and peer relationship quality variables and their interaction term to examine the robustness of our findings. Young adult intimate partner relationship variables were standardized (*z*‐score) to assist with interpretation.

### Statistical analysis

To estimate the effects of adolescent parent–child and peer relationship quality on intimate partner relationship quality in young adulthood, separate linear regression models were estimated for the ATP and TRAILS, using Generalized Estimating Equations (GEE) with an exchangeable working correlation structure (to account for clustering across waves) and a robust variance estimator. Specifically, in a series of separate models, intimate partner relationship quality in young adulthood was regressed onto adolescent: (1) parent–child relationship quality (high vs. low [reference]), (2) peer relationship quality (high vs. low [reference]), and (3) the 4‐level categorical interaction variable as a single predictor reflecting the combined effects parent–child and peer relationship quality, with the low parent and peer group serving as the reference category. Interaction terms between gender and each primary exposure were included to test whether associations differed between male and female participants. All models first adjusted for the wave of outcome measurement as a covariate to account for repeated assessments across young adulthood, before additionally adjusting for all potential confounders.

Missing data ranged from 0% to 45% in the ATP and 0% to 46% in TRAILS (full missingness reported in Table 1; highest levels of missing data driven by participants not in a relationship at a single timepoint). Multiple imputation was used to handle missing data in the inferential analyses. Fifty complete datasets were imputed, based on a multivariate normal model. Binary variables were imputed as continuous variables and then back transformed with adaptive rounding following imputation (Bernaards et al., 2007). Using Rubin's rules, estimates were obtained by pooling results across the 50 imputed data sets (Rubin, 2004).

To examine the robustness of our findings, a series of sensitivity analyses were conducted (Hernán et al., 2022). We repeated analyses: (1) examining effects across each outcome wave, (2) with adolescent parent–child and peer relationship quality reprised as continuous (*z*‐score) variables, and (3) restricting the sample to include only individuals actively in a relationship at the time of each outcome wave.

## RESULTS

### Descriptive statistics

Descriptive statistics and missing data on parent–child relationship quality, peer relationships quality, intimate partner relationship quality, and confounding variables are presented in Table 1. Comparisons of relational characteristics across the ATP and TRAILS analytic samples revealed broadly comparable results between cohorts, with similar variability in scores. For intimate partner relationship quality, despite differences in response scales (ATP: 4‐point; TRAILS: 7‐point), mean scores were consistently high in both cohorts. The average score for intimate partner relationship quality was high, as reflected by average responses ranging from “often/sometimes true” to “always/very true” for the ATP, and “somewhat agree” to “agree” for TRAILS. Adolescents in TRAILS reported higher quality parent–child relationships (72%) than those in ATP (57%). High peer relationship quality was comparable in the ATP (47%) to TRAILS (41%). In both samples, approximately one quarter of participants reported low levels of both parent–child and peer relationship quality during adolescence (ATP: 27%, TRAILS: 25%), while approximately one third reported high levels of both parent–child and peer relationship quality (ATP: 30%, TRAILS: 37%).

Adjusted associations between adolescent parent–child and peer relationship quality on intimate partner relationship quality in young adulthood are reported in Table 2. We presented the models adjusting only for outcome wave in the Supporting Information (Table S3).

**TABLE 2:** Associations between parent–child and peer relationship quality in adolescence and intimate partner relationship quality in young adulthood.

	ATP				TRAILS			
	*β*	95% CI	*p*	Gender interaction	*β*	95% CI	*p*	Gender interaction
Parent–child relationship quality	(base)			0.548	(base)			0.764
	0.12	(0.02, 0.22)	.019		0.11	(0.02, 0.20)	.014	
Peer relationship quality	(base)			0.621	(base)			0.251
	0.10	(−0.00, 0.21)	.073		0.07	(−0.01, 0.15)	.095	
Peer and parent relationship								
Low parent, low peer	(base)			0.768	(base)			0.606
High parent, low peer	0.12	(−0.02, 0.26)	.100		0.10	(−0.01, 0.20)	.076	
Low parent, high peer	0.10	(−0.06, 0.26)	.237		0.03	(−0.19, 0.25)	.779	
High parent, high peer	0.19	(0.05, 0.33)	.008		0.13	(0.03, 0.23)	.011	

*Note*: ATP = Australia; TRAILS = The Netherlands.

### Associations between adolescent parent–child and peer relationship quality and young adult intimate partner relationship quality

After accounting for the full range of confounders, we found associations between parent–child relationship quality in adolescence and intimate partner relationship quality in young adulthood that replicated across cohorts (ATP *β*_parents_ = 0.12, 95% CI = 0.02, 0.22; TRAILS *β*_parents_ = 0.11, 95% CI = 0.02, 0.20). We found a similar pattern of association with peer relationship quality that replicated across cohorts (ATP *β*_peer_ = 0.10, 95% CI = −0.00, 0.21; TRAILS *β*_peer_ = 0.07, 95% CI = −0.01, 0.15), although for both cohorts, confidence intervals marginally overlapped the null value.

### Association between adolescent parent–child and peer relationship quality interplay and young adult intimate partner relationship quality

In both cohorts, all comparisons were made against individuals who reported low parent–child and low peer relationship quality in adolescence. Compared to this reference group, those with high parent–child but low peer relationship quality showed modestly stronger young adult intimate partner relationship quality in both the ATP (*β* = 0.12, 95% CI = −0.02, 0.26) and TRAILS (*β* = 0.10, 95% CI = −0.01, 0.20), although the confidence intervals slightly overlapped the null. The strength of the association was weaker when only peer relationship quality was high (ATP: *β* = 0.10, 95% CI = −0.06, 0.26; TRAILS: *β* = 0.03, 95% CI = −0.19, 0.25). By contrast, participants with high‐quality relationships in both domains had significantly higher intimate partner relationship quality than the low–low group (ATP: *β* = 0.19, 95% CI = 0.05, 0.33; TRAILS: *β* = 0.13, 95% CI = 0.03, 0.23). This dual high‐quality group represented the strongest positive association across all categories.

### Gender differences in associations between adolescent parent–child and peer relationship quality and young adult intimate partner relationship quality

No evidence was found to suggest differences in the associations between adolescent parent–child and peer relationship quality and young adult intimate partner relationship quality for male and female participants in either ATP or TRAILS samples (*p* > .05). We report the results for males and females separately in the Supporting Information (Table S4).

### Sensitivity analyses

Several sensitivity analyses were conducted, for which the findings align with the conclusions drawn from the primary analyses. First, as presented in the Supporting Information (Table S5), the examination of effects across young adult waves revealed no significant interactions (*p* > .05), demonstrating temporal consistency. As detailed in the Supporting Information (Table S6), the direction of associations was consistent when the models utilized continuous parent–child and peer exposures, although the confidence intervals slightly overlapped the null. Comparable patterns were also observed in the intersection of parent–child and peer relationships. The interactions are visualized using the raw, unstandardized data, via contour plots depicting the average outcome scores within levels of parent–child and peer relationship quality. Patterns presented in Supporting Information (Figure S1a [ATP]; Figure S1b [TRAILS]). Finally, as presented in the Supporting Information (Table S7), restricting the analysis to only participants in a relationship at the time of each outcome wave had little effect on the effects evident in the TRAILS sample; however, smaller effect sizes were noted in the ATP.

## DISCUSSION

In this study, we utilized longitudinal data from two long‐running population‐based cohort studies in Australia and the Netherlands, to explore the independent and combined effects of parent–child and peer relationship quality in adolescence on subsequent intimate partner relationship quality in young adulthood. We used contemporary approaches (i.e., Target Trial Emulation (TTE) framework and Directed Acyclic Graphs) relevant to observational data to support causal interpretations. Our findings are consistent with a possible causal effect of adolescent parent–child and peer relationship quality on intimate partner relationship quality in young adulthood, with the strongest positive impact observed when participants, both men and women, experienced positive relationships with *both* parents and peers. Our findings, which were replicated across two distinct cohorts from two different countries, highlight the etiological significance of positive adolescent relationships, both within and outside of the home, in establishing foundations for intimate partner relationships in young adulthood.

The identified relationship between young adult intimate partner quality and both parent–child and peer relationship quality in adolescence replicates and extends earlier empirical research by providing stronger evidence of a causal effect spanning from adolescence to young adulthood (Allen et al., 2020; Kretschmer et al., 2017; Schulz et al., 2023). Additionally, these findings align with the life course and developmental cascade perspectives, which emphasize how competencies in earlier relational experiences can scaffold and feed forward to future social relationships (Masten & Cicchetti, 2010). Further, the consistency of our findings across (1) male and female participants and (2) multiple periods of young adulthood, further supports the robustness of our conclusions. The absence of gender differences suggests that while prior research has evidenced gender differences in socialization processes (Collins & Laursen, 2004; Feraco & Meneghetti, 2023; Moreau et al., 2019), they are unlikely to affect the cascading effect of relationship quality across close relationships from adolescence to young adulthood.

Notably, the finding that the strongest effects occur when both parent–child and peer relationships were higher quality underscores the combined impact of these earlier close relationships. The results of the present study are consistent with the notion that while both parent–child and peer relationships foster the development of core interpersonal skills such as communication, empathy, and conflict resolution, they do so within different social contexts and through distinct and complementary mechanisms (Furman et al., 2002; Van de Bongardt et al., 2015). This aligns with the theoretical concept of a relational ecosystem, whereby skills derived from different relationship contexts accumulate to shape later intimate partner relationship quality (Bronfenbrenner, 1979; Reis et al., 2000).

Relationships with parents, which are involuntary and governed by authority and obligation, (typically) serve as the foundational attachment bonds, which shape the internal working models that will guide future relational dynamics (Bowlby, 1982; Delgado et al., 2022). These foundational relationships, rooted in trust, communication, and connection, provide a secure working model from which individuals form expectations and behaviors in later intimate relationships, significantly influencing the success of adult intimate relationships (Mikulincer & Shaver, 2016). However, and unlike the more hierarchical structure of parent–child interactions, same‐aged peer relationships (particularly in adolescence) offer a more egalitarian context, where the emphasis is more centered on mutuality, equality, and shared decision making, which are essential for relationships outside the familial structure (Donlan et al., 2015; Laursen & Veenstra, 2021). Through these interactions, adolescents not only practice building intimacy, but also further develop the internal working models initially shaped by their parental bonds (Delgado et al., 2022), which may ultimately create a more cohesive relational framework. Through such processes, positive peer relationships have the potential to foster the development of advanced social‐cognitive skills such as emotional reciprocity and conflict resolution‐based negotiation of equality and fairness—skills that are directly transferable to later romantic relationships and critical for their success (Allen et al., 2020; Mitic et al., 2021).

As parent–child and peer relationships offer complementary developmental functions, the dual impact of these influences is likely to equip adolescents with a more refined set of interpersonal abilities across different contexts, thereby enhancing their capacity to form and maintain a cohesive working model critical for healthy intimate relationships in young adulthood. Given the combined effects observed between parent–child and peer relationships, future research would benefit from examining which specific mechanisms underpinning parent–child and peer relationship quality (e.g., working models, specific social skills, emotional regulation processes) have the greatest impact across domains of intimate partner relationship quality.

### Implications

Paying greater attention to the developmental conditions for quality intimate partner relationships is a public health priority (Holt‐Lunstad et al., 2017; World Health Organisation, 2025). Findings from our study offer valuable insights for intervention strategies by underscoring the importance of investment in both parent and peer relationships during early adolescence for preventing low intimate partner relationship quality. From a policy perspective, greater investment in early relational health interventions can be implemented in family support systems, school‐based friendship facilitation programs, and parent training workshops that collectively target the enhancement of broad relational skills. For instance, incorporating relationship education into school curricula could equip adolescents with the necessary tools to foster healthy peer interactions (Durlak et al., 2011), while parent‐focused initiatives administered through community support centers or online could enhance positive parenting practices that support emotional and social development (Sanders, 2008). Building on this study, the implementation of these evidence‐based interventions has the potential to enhance societal health by laying the groundwork for stable and fulfilling adult relationships, while also mitigating the social and economic burden associated with familial and marital breakdowns.

### Strengths and limitations

This study possesses several strengths that bolster the validity and generalizability of its findings. First, we utilized a Target Trial Emulation, an approach that supports stronger causal inferences. Second, we used long‐term prospective data, which provides a comprehensive view of the impact of close relationships in adolescence on the development of intimate relationships in young adulthood. Finally, the associations observed in our study were replicated across two cohort studies conducted in two different countries (Australia and the Netherlands). This cross‐country replication enhances the external validity and generalizability of our results.

Despite the strengths of our study, mature cohort studies are subject to important sources of selection, measurement, and confounding bias that warrant consideration (Sterne et al., 2016). While levels of missing data were reasonably low and were addressed using multiple imputation to minimize missing data bias, there is likely some selective drop‐out among the most vulnerable individuals. Additionally, while our operationalization of intimate partner relationship quality is broad and utilizes self‐report data, we recognize that this construct can be defined and measured in various, more refined ways (e.g., intimacy, commitment, and satisfaction; Joel et al., 2020). Moreover, our measures of parent–child and peer exposure were as unit‐level assessments that did not capture the nuanced heterogeneity within these relationships. For example, prior research highlights the differential effects of maternal and paternal relationships with adolescent children on subsequent outcomes in young adulthood (Macdonald et al., 2018). Additionally, analyses included only adolescents who later reported being in an intimate partner relationship during young adulthood. To build a more complete understanding of how adolescent relational experiences shape later intimate partnerships, future research should aim to examine adolescent predictors of both relational formation and relational quality. Further, although we accounted for numerous confounding variables, there remains the potential for unmeasured confounding factors that could influence our results. Future studies should aim to address these limitations to build on our findings and further advance the field. Finally, our analysis covered a large age range in young adulthood, which may not capture potential differences in relational dynamics across this period. Future research should aim to explore how these associations may vary across different stages of young adulthood as they enter new relationships and transition to more stable and committed relationships.

## CONCLUSIONS

Leveraging longitudinal data from two of the longest running studies of social and emotional development in Australia and the Netherlands, this study contributes to developmental literature on parent–child, peer and intimate partner relationships. Our results demonstrate that while adolescent parent–child and peer relationships are independently influential in the formation of intimate partner relationships, it is the combined influence that is most important for forming and maintaining healthy intimate relationships in young adulthood. These findings underscore the need for educational and community interventions during adolescence that foster positive relationships both within and outside of the home. Future research should investigate the mechanisms underlying these associations to further illustrate their impact and inform interventions aimed at fostering healthier relationships across the lifespan.

## AUTHOR CONTRIBUTIONS

**Emma M. Marshall:** Methodology; investigation; writing – review and editing; supervision. **Stephanie R. Aarsman:** Writing – review and editing. **Craig A. Olsson:** Conceptualization; data curation; funding acquisition; methodology; investigation; writing – review and editing; supervision. **Primrose Letcher:** Writing – review and editing. **Elizabeth A. Spry:** Writing – review and editing. **Khya Marabel‐Whitburn:** Conceptualization; formal analysis; methodology; investigation; writing – original draft; writing – review and editing. **Jacqui A. Macdonald:** Writing – review and editing. **Christopher J. Greenwood:** Conceptualization; formal analysis; methodology; investigation; writing – review and editing; supervision. **Tina Kretschmer:** Conceptualization; data curation; funding acquisition; formal analysis; methodology; investigation; writing – review and editing; supervision.

## FUNDING INFORMATION

ATP Funding: Data collection for the ATP study has been supported primarily through Australian grants from the Melbourne Royal Children's Hospital Research Foundation, National Health and Medical Research Council, Australian Research Council, and the Australian Institute of Family Studies. Funding for this work was supported by grants from the Australian Research Council [DP130101459; DP160103160; DP180102447] and the National Health and Medical Research Council of Australia [APP1082406]. CAO was supported by a National Health and Medical Research Council Fellowship (Investigator grant APP1175086). CG and ES were supported by a Deakin University Postdoctoral Research Fellowship. KMW was supported by the Australian Government Research Training Program Scholarship.

TRAILS Funding: TRAILS has been financially supported by various grants from the Netherlands Organization for Scientific Research, ZonMw, the Dutch Ministry of Justice, the European Science Foundation, the European Research Council, Biobanking and Biomolecular Resources Research Infrastructure, the Gratama Foundation, the Jan Dekker Foundation, the participating universities, and Accare Centre for Child and Adolescent Psychiatry. TK has received funding in form of European Research Council (ERC‐2017‐STG‐757364; ERC‐CoG‐2015‐681466). Author TK acknowledges generous funding through a European Resarch Council awarded under the Horizon Europe program (grant agreement 101087395).

## CONFLICT OF INTEREST STATEMENT

The authors have no conflicts of interest to report.

## ETHICS STATEMENT

ATP ethics approval was obtained from the Human Research Ethics Committees at the University of Melbourne, the Australian Institute of Family Studies, and/or the Royal Children's Hospital, Melbourne, 1983. TRAILs ethics approval was obtained from the Dutch Central Committee on Research Involving Human Subjects (CCMO, NL67411.042.18) and conforms to the principles laid out in the Declaration of Helsinki, 2001.

## PATIENT CONSENT STATEMENT

Participants gave informed consent to participate in the study before taking part.

## Supporting information


Appendix S1.

## Data Availability

The data that support the findings of this study are available upon request from the corresponding author. The data are not publicly available due to privacy or ethical restrictions.
