# Supplementary material for: The association between parent–child and peer relationship quality in adolescence and intimate partner relationship quality in young adulthood: A two‐cohort longitudinal investigation
Source: J Res Adolesc. 2026 May 8;36:e70184. doi: 10.1111/jora.70184 (PMC13156444; doi:10.1111/jora.70184)
Supplement: Supplementary file 1 — Appendix S1. [file JORA-36-0-s001.docx]

**The Association between Parent-Child and Peer Relationship Quality in Adolescence and Intimate Partner Relationship Quality in Young Adulthood: A Two-Cohort Longitudinal Investigation**

# Supplementary materials

| **Table S1**  *Description of Potential Confounding Factors* | | |
| --- | --- | --- |
| Variable | ATP | TRAILS |
| Parent Level |  |  |
| Country of Birth | 0=Australia, 1=Not Australia; parent report; birth | 0=The Netherlands, 1=Not The Netherlands |
| Separation | 0=No, 1=Yes; parent report | 0=No, 1=Yes; parent report |
| Age at Birth of First Child | Parent report | Parent report |
| Participant Level |  |  |
| Sex | 0=male, 2=female | 0=male, 2=female |
| Mental Health | Mean level of depression and anxiety symptoms using Short Mood and Feeling Questionnaire and the Revised Behaviour Problem Checklist Short Form; child-report; 13-14 years | Depression and anxiety symptoms using the DSM-V; child-report; 11.1 years |
| Delinquency | Frequency of two or more antisocial behaviours (e.g., damaged things in a public place, suspended/expelled from school, stolen something, physical fights); child report; 13-14 years | Withdrawn/depressed and anxious/depressed items on the Antisocial Behavior Questionnaire; child-report; 11.1 years |
| Substance use | Any tobacco use, alcohol consumption, or cannabis, amphetamine, cocaine, ecstasy, hallucinogen, or opioid; child-report; 13-14 years | Any tobacco use, alcohol consumption, or cannabis, amphetamine, cocaine, ecstasy, hallucinogen, or opioid; child-report; 13.6 years |
| Temperament | Parent’s rating of child difficulty; child-report; 4-8 months to 17-18 years | Negative affectivity items on the Early Adolescent Temperament Questionnaire (EATQ-R); child-report; 11.1 years |
| *Note*. ATP = Australia, TRAILS = The Netherlands. | | |

| **Table S2**  *Target Trial Emulation: What Is the Causal Effect of Parent-Child And Peer Relationship Quality In Adolescence On Subsequent Young Adulthood Intimate Partner Relationship Quality* | | | | | | |
| --- | --- | --- | --- | --- | --- | --- |
|  | Peer Relationship Quality | | | Parent-child Relationship Quality | | |
| Protocol component | Target trial specification | Emulation ATP | Emulation TRAILS | Target trial specification | Emulation ATP | Emulation TRAILS |
| Eligibility criteria | Adolescent (age 10-18 years) males and females from Australia and the Netherlands across the late ‘70s into the early ‘90s (1977 to 1992). | Adolescent (age 13-14 years) males and females born in Victoria, Australia in 1983. | Adolescent (age 13.6 years) males and females born in The Netherland’s in 2001. | Adolescent (age 10-18 years) males and females from Australia and across the late ‘70s into the early ‘90s (1977 to 1992). | Adolescent (age 13-14 years) males and females born in Victoria, Australia in 1983. | Adolescent (age 13.6 years) males and females born in The Netherland’s in 2001. |
|  |  |  |  |  |  |  |
| Sub-group analyses | Male and female samples. | Male and female samples. | Male and female samples. | Male and female samples. | Male and female samples. | Male and female samples. |
|  |  |  |  |  |  |  |
| Treatment strategies ^*^ | Intervention arm: intervention that improves peer relationship quality.  Comparator arm: no intervention. | Intervention: Good peer relationship quality (endorsing ‘often true’ or above on the average score of the IPPA)  Comparator: Poor peer relationship quality (endorsing less than ‘often true’ on the average score of the IPPA) | Intervention: Good peer relationship quality (endorsing ‘almost always or above on the average score of the SPF)  Comparator: Poor peer relationship quality (endorsing less than ‘almost always; on the average score of the SPF | Intervention arm: intervention that improves parent-child relationship quality.  Comparator arm: no intervention. | Intervention: Good parent relationship quality (endorsing ‘Often true’ or above on the average score of the IPPA)  Comparator: Poor parent relationship quality (endorsing less than ‘often true’ on the average score of the IPPA) | Intervention: Good parent relationship quality (endorsing ‘almost always’ or above on the average score of the SPF)  Comparator: Poor parent relationship quality (endorsing less than ‘almost always; on the average score of the SPF |
|  |  |  |  |  |  |  |
| Assignment procedures | Participants will be randomly assigned to either treatment strategy and will be aware of the strategy to which they have been assigned. | Selection of confounders.  Approach to confounder adjustment: G-computation. | Selection of confounders.  Approach to confounder adjustment: G-computation. | Participants will be randomly assigned to either treatment strategy and will be aware of the strategy to which they have been assigned. | Selection of confounders.  Approach to confounder adjustment: G-computation. | Selection of confounders.  Approach to confounder adjustment: G-computation. |
|  |  |  |  |  |  |  |
| Follow-up period | Starts: at randomisation  Ends: study drop-out, death, or young adulthood (age 19-29), whichever occurs first. | Starts: Age 13-14 years.  Ends: Age 27-28 years. | Starts: Age 13.6 years.  Ends: Age 25-26 years. | Starts: at randomisation  Ends: study drop-out, death, or young adulthood (age 19-29), whichever occurs first. | Starts: Age 13-14 years.  Ends: Age 27-28 years. | Starts: Age 13.6 years.  Ends: Age 25-26 years. |
|  |  |  |  |  |  |  |
| Outcome ^*^ | Improved intimate partner relationship quality (age 19-28 years). | Closeness and conflict (Braiker & Kelley) | Social Production Functions (SPF) Questionnaire | Improved intimate partner relationship quality (age 19-28 years). | Closeness and conflict (Braiker & Kelley) | Social Production Functions (SPF) Questionnaire |
|  |  |  |  |  |  |  |
| Causal contrast | Comparator arm versus intervention arm: Risk Ratio. | | | | |  |
| *Note*. ATP = Australia, TRAILS = The Netherlands. | | | | | |  |

| **Table S3** *Unadjusted Associations Between Adolescent Parent-Child and Peer Relationship Quality and Young Adult Intimate Partner Relationship Quality* | | | | | | |
| --- | --- | --- | --- | --- | --- | --- |
|  | ATP | | | TRAILS | | |
|  | b | 95% CI | p | b | 95% CI | p |
| Parent-Child Relationship Quality | (base) |  |  | (base) |  |  |
|  | 0.15 | (0.04, 0.25) | 0.003 | 0.10 | (0.14, 0.20) | 0.023 |
| Peer Relationship Quality | (base) |  |  | (base) |  |  |
|  | 0.17 | (0.06, 0.27) | 0.003 | 0.09 | (0.02, 0.18) | 0.018 |
| Parent-Child and Peer Relationship Quality | |  |  |  |  |  |
| Low Parent, Low Peer | (base) |  |  | (base) |  |  |
| High Parent, Low Peer | 0.15 | (0.01, 0.30) | 0.031 | 0.07 | (-0.04, 0.18) | 0.201 |
| Low Parent, High Peer | 0.18 | (0.01, 0.35) | 0.033 | 0.04 | (-0.18, 0.27) | 0.719 |
| High Parent, High Peer | 0.28 | (0.13, 0.42) | 0.000 | 0.15 | (0.04, 0.25) | 0.005 |
| *Note*. ATP = Australia, TRAILS = The Netherlands. | | | | | | |

| **Table S4** *Associations Between Parent-Child And Peer Relationship Quality In Adolescence and Intimate Partner Relationship Quality In Young Adulthood By Gender* | | | | | | |
| --- | --- | --- | --- | --- | --- | --- |
|  | ATP | | | TRAILS | | |
|  | b | 95 CI | p | b | 95 CI | p |
|  | Male sample | | | | | |
| Parent-Child Relationship Quality | (base) |  |  | (base) |  |  |
|  | 0.17 | (-0.01, 0.34) | 0.061 | 0.11 | (-0.03,0.26) | 0.129 |
| Peer Relationship Quality | (base) |  |  | (base) |  |  |
|  | 0.07 | (-0.12, 0.26) | 0.474 | 0.15 | (0.01,0.29) | 0.041 |
| Parent-Child and Peer Relationship Quality | |  |  |  |  |  |
| Low Parent, Low Peer | (base) |  |  | (base) |  |  |
| High Parent, Low Peer | 0.16 | (-0.06, 0.36) | 0.157 | 0.07 | (-0.09,0.23) | 0.378 |
| Low Parent, High Peer | 0.00 | (-0.35, 0.35) | 0.981 | 0.15 | (-0.23,0.53) | 0.46 |
| High Parent, High Peer | 0.21 | (-0.04, 0.45) | 0.102 | 0.19 | (0.01,0.37) | 0.031 |
|  | Female Sample | | | | | |
| Parent-Child Relationship Quality | (base) |  |  | (base) |  |  |
|  | 0.08 | (-0.04, 0.21) | 0.192 | 0.10 | (-0.00, 0.21) | 0.064 |
| Peer Relationship Quality | (base) |  |  | (base) |  |  |
|  | 0.11 | (-0.02, 0.24) | 0.101 | 0.01 | (-0.08, 0.11) | 0.767 |
| Parent-Child and Peer Relationship Quality | |  |  |  |  |  |
| Low Parent, Low Peer | (base) |  |  | (base) |  |  |
| High Parent, Low Peer | 0.09 | (-0.12, 0.29) | 0.412 | 0.11 | (-0.02, 0.24) | 0.088 |
| Low Parent, High Peer | 0.11 | (-0.14, 0.26) | 0.232 | -0.01 | (-0.29, 0.27) | 0.956 |
| High Parent, High Peer | 0.17 | (0.00, 0.35) | 0.05 | 0.09 | (-0.03, 0.21) | 0.155 |
| *Note*. ATP = Australia, TRAILS = The Netherlands. | | |  |  |  |  |

| **Table S5**  *ATP and TRAILS Parent-Child, Peer and Intimate Partner Relationship Quality Interaction with Wave* | | | | | | | | | | |
| --- | --- | --- | --- | --- | --- | --- | --- | --- | --- | --- |
|  | ATP | | | | | | | | |  |
|  | b | 95% CI | p | b | 95% CI | p | b | 95% CI | p | Wave Interaction |
|  | 19-20 years | | | 23-24 years | | | 27-28 years | | |  |
| Parent-Child Relationship Quality | (base) |  |  | (base) |  |  | (base) |  |  | 0.328 |
|  | 0.14 | (-0.04, 0.33) | 0.143 | 0.18 | (0.03, 0.32) | 0.018 | 0.04 | (-0.08, 0.17) | 0.538 |  |
| Peer Relationship Quality | (base) |  |  | (base) |  |  | (base) |  |  | 0.519 |
|  | 0.12 | (-0.09, 0.32) | 0.254 | 0.15 | (-0.00, 0.31) | 0.056 | 0.03 | (-0.10, 0.16) | 0.688 |  |
| Parent-Child and Peer Relationship Quality | | |  |  |  |  |  |  |  |  |
| Low Parent, Low Peer | (base) |  |  | (base) |  |  | (base) |  |  | 0.541 |
| High Parent, Low Peer | 0.19 | (-0.06, 0.43) | 0.132 | 0.18 | (-0.01, 0.39) | 0.071 | -0.01 | (-0.18, 0.17) | 0.955 |  |
| Low Parent, High Peer | 0.18 | (-0.06, 0.44) | 0.193 | 0.16 | (-0.06, 0.39) | 0.165 | -0.04 | (-0.22, 0.16) | 0.721 |  |
| High Parent, High Peer | 0.23 | (-0.04, 0.49) | 0.099 | 0.28 | (0.08, 0.28) | 0.006 | 0.06 | (-0.11, 0.23) | 0.532 |  |
|  | TRAILS | | | | | | | | |  |
|  | 19.1 years | | | 22.3 years | | | 25-26years | | |  |
| Parent-Child Relationship Quality | (base) |  |  | (base) |  |  | (base) |  |  | 0.536 |
|  | 0.06 | (-0.11, 0.23) | 0.478 | 0.17 | (0.03, 0.31) | 0.015 | 0.10 | (-0.01, 0.21) | 0.067 |  |
| Peer Relationship Quality | (base) |  |  | (base) |  |  | (base) |  |  | 0.869 |
|  | 0.06 | (-0.08, 0.22) | 0.374 | 0.09 | (-0.03, 0.21) | 0.157 | 0.05 | (-0.06, 0.15) | 0.357 |  |
| Parent-Child and Peer Relationship Quality | | |  |  |  |  |  |  |  |  |
| Low Parent, Low Peer | (base) |  |  | (base) |  |  | (base) |  | (base) | 0.794 |
| High Parent, Low Peer | 0.00 | (-0.19, 0.19) | 0.195 | 0.96 | (0.00, 0.31) | 0.05 | 0.13 | (-0.01, 0.26) | 0.070 |  |
| Low Parent, High Peer | -0.10 | (-0.49, 0.30) | 0.633 | 0.05 | (-0.30, 0.40) | 0.79 | 0.14 | (-0.12, 0.41) | 0.292 |  |
| High Parent, High Peer | 0.09 | (-0.10, 0.27) | 0.363 | 0.19 | (0.03, 0.35) | 0.019 | 0.12 | (-0.01, 0.25) | 0.069 |  |
| *Note*. ATP = Australia, TRAILS = The Netherlands. | | | | | | | |  |  |  |

| **Table S6** *Associations between parent-child and peer relationship quality in adolescence and intimate partner relationship quality in young adulthood, using continuous measurement of exposure* | | | | | | | | |
| --- | --- | --- | --- | --- | --- | --- | --- | --- |
|  | ATP | | | | TRAILS | | | |
|  | b | 95% CI | p | Wave Interaction | b | 95% CI | p | Wave Interaction |
| Parent-Child Relationship Quality | 0.08 | (0.02, 0.14) | 0.006 | 0.168 | 0.06 | (0.02, 0.10) | 0.006 | 0.409 |
| Peer Relationship Quality | 0.08 | (0.01, 0.14) | 0.020 | 0.077 | 0.03 | (-0.01, 0.07) | 0.116 | 0.711 |
| Parent-Child X Peer Relationship Quality | 0.06 | (-0.01, 0.17) | 0.172 |  | 0.01 | (-0.01, 0.11) | 0.182 |  |
| *Note*. ATP = Australia, TRAILS = The Netherlands. | | | | | | | |  |

| **Table S7** Associations between parent-child and peer relationship quality in adolescence and intimate partner relationship quality in young adulthood, including only participants in a relationship | | | | | | | | |
| --- | --- | --- | --- | --- | --- | --- | --- | --- |
|  | **ATP** | | | | **TRAILS** | | | |
|  | b | 95% CI | p | Wave Interaction | b | 95% CI | p | Wave Interaction |
| Parent-Child Relationship Quality | (base) |  |  |  | (base) |  |  |  |
|  | 0.05 | (0.00, 0.11) | 0.066 | 0.475 | 0.12 | (0.03, 0.21) | 0.01 | 0.614 |
| Peer Relationship Quality | (base) |  |  |  | (base) |  |  |  |
|  | 0.04 | (-0.02, 0.1) | 0.206 | 0.341 | 0.07 | (-0.01, 0.15) | 0.105 | 0.852 |
| Parent-Child and Peer Relationship Quality | | |  |  |  |  |  |  |
| Low Parent, Low Peer | (base) |  |  | 0.326 | (base) |  |  | 0.880 |
| High Parent, Low Peer | 0.05 | (-0.03, 0.13) | 0.194 |  | 0.11 | (0.00, 0.22) | 0.043 |  |
| Low Parent, High Peer | 0.04 | (-0.05, 0.13) | 0.412 |  | 0.05 | (-0.19, 0.30) | 0.688 |  |
| High Parent, High Peer | 0.08 | (0.00, 0.16) | 0.045 |  | 0.14 | (0.03, 0.25) | 0.009 |  |
| *Note*. ATP = Australia, TRAILS = The Netherlands. | | | | | | | |  |

| **Figure S1a**  *ATP interplay between parent-child and peer relationship quality in adolescence and intimate partner relationship quality in young adulthood, using full sample and continuous exposure* | **Figure S1b**  *TRAILS interplay between parent-child and peer relationship quality in adolescence and intimate partner relationship quality in young adulthood, using full sample and continuous exposure* |
| --- | --- |
| 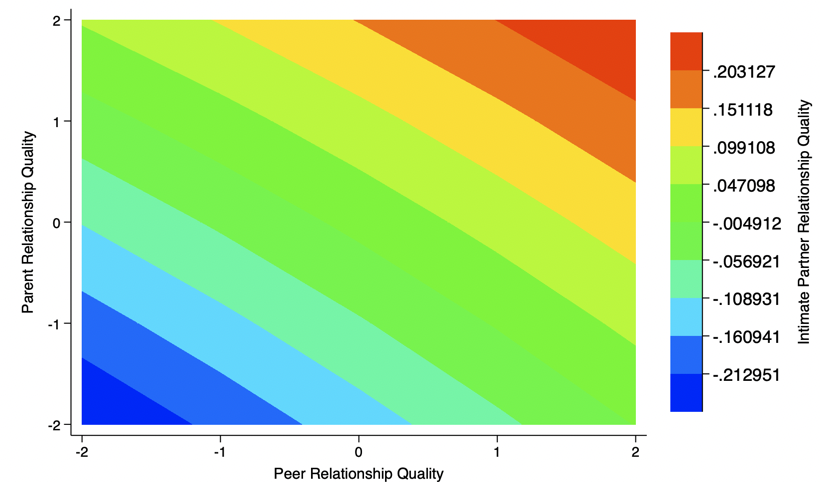 | 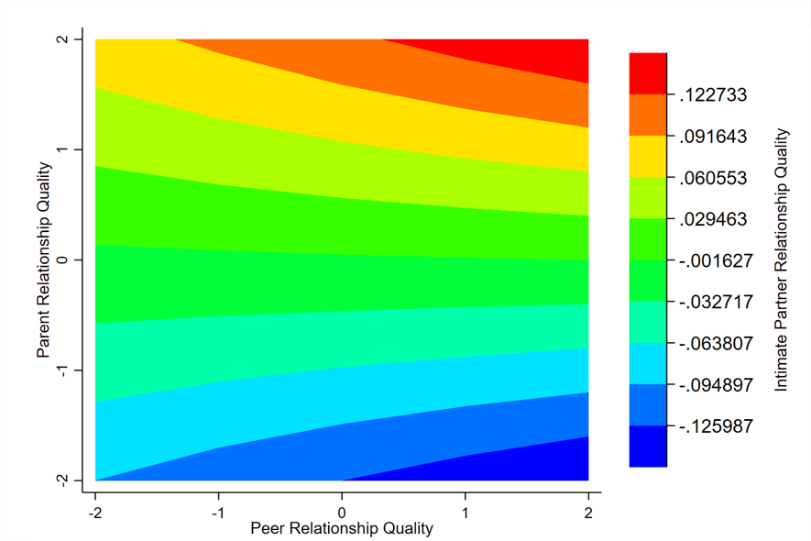 |
